# Bacterial Resistance to Penicillin G by Decreased Affinity of Penicillin-Binding Proteins: A Mathematical Model

**DOI:** 10.3201/eid0904.020213

**Published:** 2003-04

**Authors:** L. Temime, P.Y. Boëlle, P. Courvalin, D. Guillemot

**Affiliations:** * Institute National de la Santé et de la Recherdhé, Paris, France; † Unité des Agents Antibactériens, Institut Pasteur, Paris, France

**Keywords:** antibiotic resistance, mathematical models, *Streptococcus pneumonia*, *Neisseria meningitidis*, penicillin G, microbiology, epidemiology, selection, transmission, perspective

## Abstract

*Streptococcus pneumoniae* and *Neisseria meningitidis* have very similar mechanisms of resistance to penicillin G. Although penicillin resistance is now common in *S. pneumoniae*, it is still rare in *N. meningitidis*. Using a mathematical model, we studied determinants of this difference and attempted to anticipate trends in meningococcal resistance to penicillin G. The model predicted that pneumococcal resistance in a population similar to that of France might emerge after 20 years of widespread use of β-lactam antibiotics; this period may vary from 10 to 30 years. The distribution of resistance levels became bimodal with time, a pattern that has been observed worldwide. The model suggests that simple differences in the natural history of colonization, interhuman contact, and exposure to β-lactam antibiotics explain major differences in the epidemiology of resistance of *S. pneumoniae* and *N. meningitidis*.

*Streptococcus pneumoniae* and *Neisseria meningitidis* have very similar mechanisms of resistance to penicillin G, which are mediated by the decreased affinity of penicillin-binding proteins (PBPs) (*1*–*3*). However, the epidemiology of resistance of these two bacteria exhibit very different patterns.

*S. pneumoniae* strains with decreased susceptibility have been found frequently over the last decade, and most of them now have a penicillin G MIC greater than 2 µg/mL (*4*,*5*). By contrast, for *N. meningitidis*, reports of high levels of resistance remain anecdotal, even though decreased susceptibility has become more frequent (*6*).

Pneumococcal resistance has already given rise to therapeutic problems (*7*). Because meningococcal infections are highly lethal, meningococcal resistance is a major concern. Therefore, better understanding of *S. pneumoniae* resistance selection and establishing whether meningococcal resistance could increase are important.

In both *S. pneumoniae* and *N. meningitidis*, humans are the only reservoir, and asymptomatic colonization is frequent. However, the natural history of colonization differs in these two bacterial species. The average colonization duration of *S. pneumoniae* is approximately 2 to 3 months (*8*), whereas duration is approximately 10 months for *N. meningitidis* (*9*). Asymptomatic carriage of *S. pneumoniae* peaks during the first 2 years of life and then gradually declines (*10*). By contrast, carriage of *N. meningitidis* peaks in young adults (*9*), which implies a difference in antibiotic exposure and therefore in the selection pressure borne by these bacteria, as young children are treated more frequently than young adults.

Mathematical models can be used to explain how these factors interact in the selection of resistant strains and lead to different trends. Models of transmission have been developed to examine how antibiotic use affects the colonization rate of resistant commensal bacteria in human populations (*11*), to examine treatment protocols for resistance prevention (*12*), and to predict future trends (*13*). However, these models are based on a priori hypotheses which, in general, assume that the impact of antibiotic exposure does not differ according to the mechanism of resistance and do not consider the particular natural history of the colonization of the bacterial species.

We describe a mathematical model of the emergence and diffusion of bacterial resistance in the community. This model is specific to the mechanism of resistance to penicillin G common to *S. pneumoniae* and *N. meningitidis* and mediated by the decrease in affinity of their PBPs. The model also takes into account the natural histories of colonization of the two bacteria.

Using this model, we first explored a case of *S. pneumoniae* and validated our predictions by using independently obtained epidemiologic data. Next, we studied *N. meningitidis* to anticipate its trends in penicillin G–resistance selection according to antibiotic exposure.

## Materials and Methods

### Microbiologic Background and Hypotheses

β-lactam antibiotics, such as penicillin G, bind to PBPs in the bacterial cell wall. In both *S. pneumoniae* and *N. meningitidis*, the main mechanism of penicillin G resistance is mediated by the alteration of these penicillin target enzymes. The genetic events leading to reduced affinity for penicillin G are point mutations. These mutations confer slight increases in resistance and acquisition by transformation from other commensal species of the pharynx of intragenic sequences. This process leads to the synthesis of mosaic PBPs and confers higher levels of resistance (*14*–*16*).

By convention, the decrease in susceptibility to penicillin G is defined by an MIC >0.1 µg/mL and resistance by an MIC >2 µg/mL (*5*). In the laboratory, the MIC is determined by successive dilutions and presented on a log_2_ scale. However, genetic events may lead to a difference of less than a log_2_ unity between two MICs; for example, an increase from 0.04 to 0.06 µg/mL was reported by Hakenbeck et al. (*17*).

To take this progressive evolution into account, we theorized that each bacterial generation provided an opportunity for an increase in resistance. More precisely, we randomly selected for each new generation an increase in the bacterial MIC, defined as *d*, according to the seminormal law:

*f*_Δ_(*d*) = 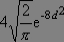


With this formulation, a high probability exists that either no genetic event occurs or that such an event will only convey a small increase in MIC, although an increase to any resistance level as a result of one genetic event remains possible. In particular, a detectable genetic event, that is, an event conveying an increase in the MIC of more than one log_2_ unity, will occur with a frequency of 10^-7^, which is consistent with previous in vitro observations (*18*).

Bacteria colonize human hosts in large quantities. Therefore, even though the occurrence of genetic events remains possible for each bacterium, competition makes it unlikely that a host’s bacterial population will suddenly be replaced by genetically altered strains. However, in the presence of antibiotic treatment, selective killing of the susceptible bacterial population may allow replacement by a less susceptible population. We therefore assumed that genetic events leading to effective bacterial replacement were only possible in treated persons. We represented the entire bacterial population of a colonized host by one MIC.

### Model Description

**Figure 1:**
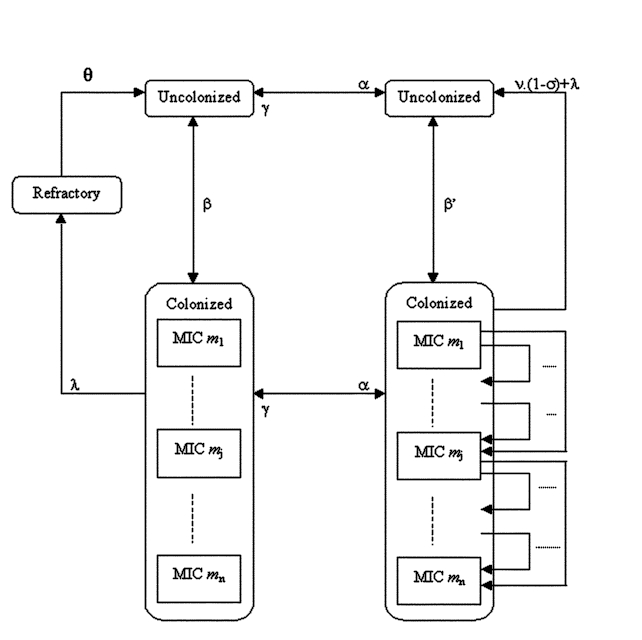
Model structure of the emergence and transmission of penicillin G resistance in *Streptococcus pneumoniae* and *Neisseria meningitidis*.

To reproduce the selection and spread of resistant bacteria in the community through interhuman transmission, we divided the human population under study into several groups or “compartments” (Figure 1). Each compartment was composed of persons with the same characteristics regarding colonization and antibiotic treatment. The colonized compartments were split into several subcompartments according to MIC.

Uncolonized persons are colonized after an infectious contact with colonized persons at rate β. In the absence of antibiotic treatment, the persons are then naturally decolonized after a time 1/λ, regardless of MIC. This period, called the duration of carriage, is followed by a refractory period of duration 1/θ, during which these persons cannot be colonized again.

With antibiotic treatment, bacterial colonization is cleared with a probability σ. In persons in whom colonization is not eliminated, bacteria with a mutation towards a higher MIC may replace the original strains.

Finally, progression from the untreated category to the treated category occurs at the start of an antimicrobial treatment, which takes place with a frequency α, and the return to the untreated category occurs when the treatment comes to an end, after an average duration of 1/γ

### Parameters

The mean duration of carriage is reportedly 2.2 months for *S. pneumoniae* and 10 months for *N. meningitidis* (*8*,*9*). Although temporary systemic immunization has occurred after colonization by these bacteria (*19*), the exact duration of this refractory phase is not clear. We chose a duration of 2 weeks and investigated the range from 4 days to 2 months.

In studies of treatment patterns in France, the average duration of antibiotic treatment was 8 days (*20*,*21*) and the frequency of treatment changed with age. Young children may be treated several times a year with penicillin G, while healthy adults are only treated once every 4 to 5 years on average. The colonization frequency also changes with age, corresponding to colonization peaks in children for *S.* *pneumoniae* and in young adults for *N. meningitidis*. In our model, the population was not structured by age, but we wanted to reflect these heterogeneities. We therefore calculated effective treatment frequencies by weighting observed frequencies of treatment with probabilities of colonization according to age, which led us to study the effects of one treatment every 2 years for *S.* *pneumoniae* and one treatment every 3 years for *N. meningitidis*.

We assumed that, with treatment, all bacteria were submitted to the same concentration of antibiotics; therefore, we considered the probability of decolonization after treatment as a function of MIC only. A commonly used model for the effect of an antibiotic on bacteria with a given MIC according to drug concentration is the saturating model (*13*). By analogy, we expressed the effect of a given antibiotic concentration in terms of the probability of nondecolonization as a function of the MIC *m*, by

σ(*m*) = 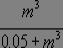.

We chose a constant infectious contact rate β in the absence of treatment. We adjusted the value of β so that the predicted proportion of carriers matched the observed values of 45% in the case of *S. pneumoniae* (*10*) and of 10% in the case of *N. meningitidis* (*9*). This gave β=0.23 weeks^-1^ person^-1^ for *S. pneumoniae* and β=0.026 weeks^-1^ person^-1^ for *N. meningitidis*. However, we assumed that a treated person had a better chance of being colonized after a contact than an untreated person if the bacteria involved had a high MIC and that colonization was less probable with susceptible bacteria. We also adopted the following sigmoidal function of the MIC *m* for the contact rate in the presence of treatment

β’(*m*) = 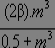.

The values of the model’s parameters are specified in Table 1. Model simulations are described in the Appendix.

**Table 1:** Model parameters and their values (8,21)

Parameters (at MIC *m*)		Pneumococci	Meningococci
Treatment duration	1/γ	8 d	8 d
Weighted frequency of treatment	α	1 / 2 y	1 / 3 y
Refractory phase duration	1/θ	2 wk	2 wk
Carriage duration	1/λ	2.2 mo	10 mo
Time before antibiotic action	1/ν	4 d	4 d
Contact rate (absence of treatment)	β	0.23 wk^-1^person^-1^	0.026 wk^-1^person^-1^
Contact rate (presence of treatment)	β´(*m*)	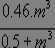	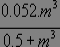
Nondecolonization probability after treatment	σ (*m*)	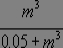	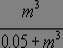
MIC increase after a genetic event	F (*m*)	Randomly selected from a seminormal law	

### *S. pneumoniae* Historical Data in France

To validate the predictions of the model for changes in resistance, we used data on *S. pneumoniae* reported to the French National Reference Center (NRC) for pneumococci (*22*). In short, 40–50 centers throughout France collected and sent *S. pneumoniae* strains to the NRC. Each year approximately 2,000 strains were typed and evaluated for susceptibility to various antibiotics. We used data from 1987 to 1997 and looked at changes in the distribution of penicillin G MICs over time.

## Results

### Predictions for *S. pneumoniae*

#### Emergence

**Figure 2:**
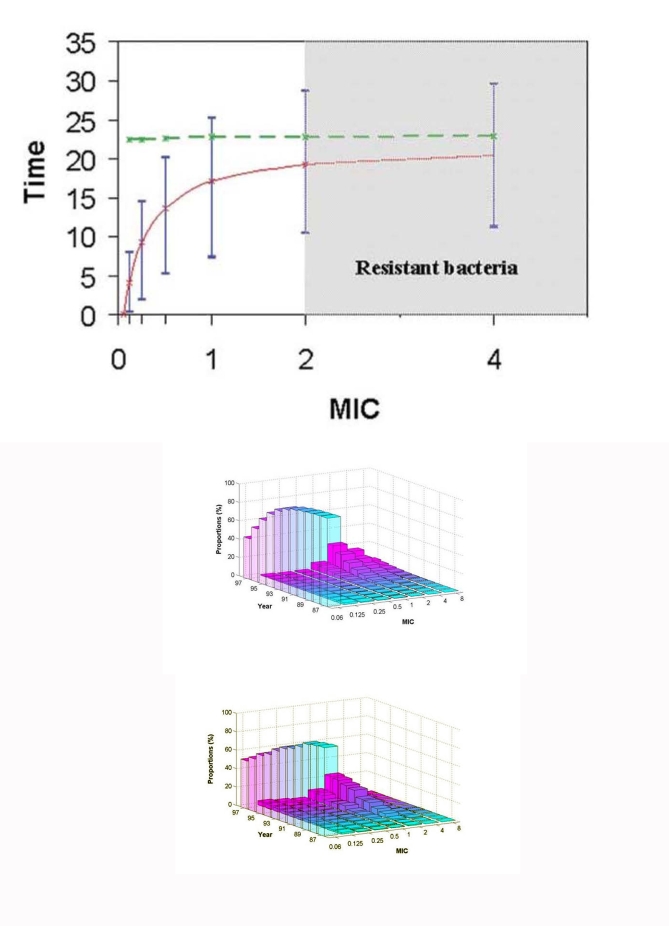
(a) Time to emergence of the first *Streptococcus pneumoniae* with a given MIC (full line) and time required for 20% of the bacterial population to reach this MIC (dotted line), starting from an all-susceptible pneumococcal population. Error bars correspond to stochastic variations in the model simulations (10th and 90th percentiles based on 100 simulations). (b) Simulated and (c) observed changes with time since 1987 in the distribution of resistance levels in the pneumococcal population in France. Observed data are taken from the Centre National de Référence des Pneumocoques (*4*).

By applying the model to a population in which all the pneumococci were initially susceptible to penicillin G (MIC < 0.06 µg/mL), we determined the time of emergence of the first strains with decreased susceptibility (MIC = 0.125, 0.25, 0.5, and 1 µg/mL), as well as the first resistant strains (MIC = 2 µg/mL) and highly resistant strains (MIC = 4 µg/mL). The model also provided information on the variability of these emergence times (Figure 2a). In particular, the model predicted the emergence of high resistance levels (MIC >2 µg/mL) after approximately 20 years of antibiotic use.

At low resistance levels, the mean time to emergence depended strongly on MIC. At higher levels (MIC >1 µg/mL), however, it reached a plateau, as the lag between the emergence of two successive levels decreased. The variability of these estimated times to emergence was marked, ranging from 10 to 30 years when starting from the same situation for the emergence of a strain with MIC 2 µg/mL.

We sought to clarify the relationship between the time required for a strain with a given MIC to be selected and the time in which the strain spreads to a large portion of the population. We determined the time at which 20% of the colonized population would be carrying strains with MIC levels ranging from >0.125 to 4 µg/mL (Figure 2a). This time appeared to depend very little on the MIC, even at low resistance levels, in contrast to the time to emergence, which began with a large increase with MIC. However, both times displayed comparable variability (data not shown).

### Transmission

We applied the model to a population in which resistance had already emerged, so that most pneumococcal strains were still susceptible to penicillin G, but some strains had high MICs. This pattern corresponds to the situation in France around 1987 (*22*). Figure 2b illustrates the model’s predictions for the evolution with distribution time of pneumococcal strains according to their MIC. In particular, after a few years, this distribution acquired a bimodal shape, with a peak for susceptible bacteria and another for resistant bacteria.

Figure 2c shows the evolution of pneumococcal resistance to penicillin G during 1987 to 1997, as observed by the French National Reference Center for Pneumococci (*4*). In 1997, the distribution of resistance levels, which was initially unimodal, exhibited a bimodal shape, with a peak for susceptible bacteria and another for resistant bacteria. These levels are consistent with the model’s predictions (Figure 2b).

### Predictions for *N. meningitidis*

**Figure 3:**
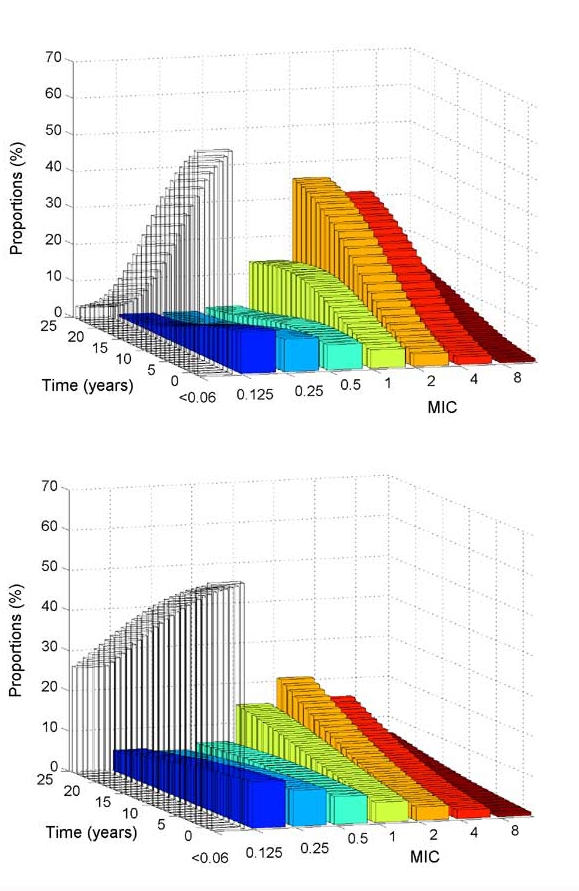
Simulated changes with time in the distribution of resistance levels in the meningococcal population, starting from a situation close to that of France in 2001, under (a) constant antibiotic treatment conditions (1 treatment/3 y) and (b) a frequency of treatment reduced by half (1 tretatment/6 y).

With approximately 30% of strains with reduced susceptibility to penicillin G, we used the same model to predict changes in the distribution of meningococcal MIC levels. Meningococcal resistance seemed to increase in the same way as pneumococcal resistance and also exhibited a bimodal distribution of MIC levels (Figure 3a). However, change was slower in this case because of reduced frequency of treatment in the population concerned and reduced transmissibility.

We studied a situation in which intervention would reduce the frequency of treatment by half (Figure 3b). Even under this reduced antibiotic pressure, high levels of resistance eventually appeared but with a delay of approximately 15 years.

## Discussion

In this study, we developed a mathematical model of the emergence and spread of penicillin G–resistant bacteria in the community that was specific to a resistance mechanism common to *S. pneumoniae* and *N. meningitidis*. The model shows that differences in the natural history of colonization, contact, and treatment rates can account for the differences in the epidemiology of the resistance of these two bacterial species.

Figure 2a highlights the difference between the isolation of a strain of reduced susceptibility and its spread in the community. A strain with a low resistance level does not have enough selective advantage to assure its persistence in the population. Therefore, this strain will probably disappear before a genetic event causes an increase of its MIC. For example, the large difference between the mean times of emergence of a strain with an MIC of 0.125 µg/mL and a strain with an MIC of 0.5 µg/mL corresponds to several successive processes of the emergence and elimination of strains with an MIC <0.5 µg/mL. On the contrary, at resistance levels greater than 1 µg/mL, the emergence of a strain frequently leads to its spread in the community and the prompt emergence of strains with higher resistance levels. After the first emergence of such a strain, it may take a few years to spread to 20% of the colonized population with an MIC of 2 µg/mL.

One major finding was the variability of the time to selection of bacteria with a given MIC. For example, starting from an all-susceptible bacterial population, a strain of *S. pneumoniae* with an MIC >2 µg/mL could be selected as soon as 10 years after the start of antibiotic use but also as late as 30 years later. Furthermore, this finding suggests that the absence of emergence after 30 years is unlikely, which is consistent with observations (e.g., the first penicillin G-resistant *S. pneumoniae* strains worldwide appeared around 1970, while penicillin G had been commonly used since 1950) (*22*).

The model predicted an increase in pneumococcal resistance leading to bimodality of MIC levels. This increase was also noted in the French data (*4*), as well as in other studies (*23*–*25*). Good agreement exists between the predicted and observed values (Figure 2).

A prediction of the model is that resistance of *N. meningitidis* will probably increase, although slowly, even if antibiotic pressure were reduced (Figure 3b). Several parameters chosen were derived from direct measures in the community or in vitro, but others required indirect evaluations. We performed a sensitivity analysis using the Latin Hypercube sampling technique (*26*). This technique showed that for predicting the percentage of resistant bacteria, the frequency of treatment was the most critical parameter (positively linked, Table 2), followed by the carriage duration (negatively linked), the treatment duration (positively linked), and the contact rate (positively linked). Also, a reasonable range of values for the duration of the refractory phase has little effect on model outcomes. Likewise, the choice of the constants and of the exponent of *m* in the probability of nondecolonization after treatment σ(m) and in the contact rate β´(m) do not have critical effects.

**Table 2:** Sensitivity analysis of the model^a^

Parameters		PRCC
Weighted frequency of treatment	α	0.981651
(Carriage duration) ^–1^	λ	0.672063
(Treatment duration) ^–1^	γ	-0.472343
Contact rate (absence of treatment)	β	0.392559

^a^Key factors that increase (Partial Rank Correlation Coefficient (PRCC>0) or decrease (PRCC<0) the prevalence of penicillin-resistant *Streptococcus pneumoniae* after 10, starting from an all-susceptible population.

The following simplifications were adopted in the model. First, rather than considering explicitly the changes in treatment frequencies with age, we used a treatment frequency averaged over age. Second, we only considered resistance caused by decreased affinity to PBPs, although other mechanisms may contribute to increase resistance (*27*,*28*), and we used the same mathematical description for all genetic events leading to resistance, i.e., point mutations and genetic material transfer (*29*) because this is supported by experimental observations (*30*). The very shape of the distribution used to model these increase in MIC did not alter the predictions, because selection of resistant strains in the community arose primarily by interhuman transmission. Finally, we did not include a fitness cost for resistant *S. pneumoniae* or *N. meningitidis*, although it has been found in other bacterial species (*31*), with the consequence that resistance progression may eventually spread faster than predicted here.

Insofar as our model takes into account both the natural history of colonization and the resistance mechanism of the bacteria considered (Appendix), the model is more realistic than general models such as those previously developed to obtain a general view of resistance (*32*). However, several aspects of the model could still be more complex to address specific problems, even though a certain level of simplification remains compulsory in a model. For instance, several serotypes of both *S. pneumoniae* and *N. meningitidis* cocirculate in the community. Differences exist in the transmissibility and duration of carriage of these bacteria, depending on their serotype; these differences could impact on resistance selection (*9*). Taking two or more bacterial serogroups into account instead of one would therefore be worth considering.

Moreover, we considered treatment with β-lactam antibiotics, whereas several antibiotics are widely used in the community. Penicillin G–resistant *S. pneumoniae* tends to be increasingly multidrug resistant (*4*). Taking this resistance into account may increase the impact of antibiotic exposure and therefore accelerate the changes predicted by the model. The general framework we described could be adapted to the study of other bacteria, provided the parameter values were chosen to reflect the natural history of colonization and the way in which the treatment failure probability σ (*m*) depends on the MIC *m*.

Finally, recently developed polysaccharide-protein conjugate vaccines have been shown to protect persons against symptomatic and asymptomatic colonization by *S. pneumoniae* or *N. meningitidis* (*33*,*34*). This protection is specific to the serotypes included in the vaccines. Our model could evaluate the impact of the use of such vaccines. Strategies for vaccination against *S. pneumoniae* or *N. meningitidis* may differ widely; therefore, vaccination for all children is recommended for *S. pneumoniae* (*35*), whereas targeted vaccination campaigns are more often conducted for *N. meningitidis* (*36*,*37*).

## Supplementary Material

Appendix Numerical Treatment of the Model
