# Supplementary material for: Bacterial Resistance to Penicillin G by Decreased Affinity of Penicillin-Binding Proteins: A Mathematical Model
Source: Emerg Infect Dis. 2003 Apr;9(4):411–7. doi: 10.3201/eid0904.020213 (PMC2957969; doi:10.3201/eid0904.020213)
Supplement: Appendix — Numerical Treatment of the Model [file 02-0213_app-s1.pdf]

Appendix

Numerical Treatment of the Model

Classically, compartmental models are studied through a set of differential equations, which allows the computing of deterministic predictions. However, it is also possible to consider the transitions between compartments as random phenomena, to which a probability can be associated. This latter approach, called stochastic, offers the advantage of being more realistic than the classical deterministic version, as it takes into account the biological variability of the system and enables its measuring. We used both a deterministic and a stochastic version of the model in this study.

Deterministic Model

The equations used are as follows

$$\left\{\begin{array}{ll}
\frac{dU_{np}}{dt}(t) & = -\alpha U_{np}(t) + \gamma V_{np}(t) - \beta U_{np}(t)[\int_0^\infty (U_p(c,t) + V_p(c,t))dc] + \theta U_{ref}(t) \\
\frac{dU_p}{dt}(m,t) & = -(\alpha + \lambda)U_p(m,t) + \gamma V_p(m,t) + \beta U_{np}(t)\,[U_p(m,t) + V_p(m,t)] \\
\frac{dU_{ref}}{dt}(t) & = \lambda \int_0^\infty U_p(c,t)dc - \theta U_{ref}(t) \\
\frac{dV_{np}}{dt}(t) & = \alpha U_{np}(t) - \gamma V_{np}(t) - V_{np}(t) \int_0^\infty \beta'(c)\,[U_p(c,t) + V_p(c,t)]dc \\
 & +\lambda \int_0^\infty V_p(c,t)dc + \nu \int_0^\infty [1 - \sigma(c)]V_p(c,t)dc \\
\frac{dV_p}{dt}(m,t) & = \alpha U_p(m,t) - (\gamma + \lambda + \nu(1 - \sigma(m)))V_p(m,t) + \beta'(m)V_{np}(t)(U_p(m,t) + V_p(m,t)) \\
 & -\nu\sigma(m)V_p(m,t) + \nu \int_0^m \sigma(c)\mathcal{F}(m - c)V_p(c,t)dc
\end{array}\right.$$

where at time t, $U_{np}(t)$ is the proportion of uncolonized untreated hosts in the population, $U_{ref}(t)$ the proportion of untreated hosts in a refractory phase, $V_{np}(t)$ the proportion of uncolonized treated hosts, $U_p(m,t)$ the density of untreated hosts colonized with bacteria with MIC *m*, and $V_p(m,t)$ the density of treated hosts colonized with bacteria with MIC *m* (38).

Stochastic Model

Interested readers may contact the first author for technical details about the simulations that were performed by using Gillespie's Direct Method (39).
